# Consenting for Spinal Cord Stimulation – the Pitfalls and Solution

**DOI:** 10.1007/s11916-024-01349-w

**Published:** 2025-02-01

**Authors:** Vivek Mehta, Rajesh Munglani, Giles Eyre, Gaurav Bajaj, Alaa Abd-Elsayed, Kavita Poply

**Affiliations:** 1 Barts Health NHS Trust, Pain Research Centre, London, UK; 2 St Thomas Hospital, London, UK; 3 Chambers of Jacob Levy KC, London, UK; 4 University of Wisconsin-Madison, Madison, WI USA; 5 Queen Mary University, London, UK

**Keywords:** Consent, Spinal cord stimulation, Claims, Complications, Pitfalls, Solutions, Chronic pain

## Abstract

**Purpose of Review:**

To discuss the importance of the consenting process for patients undergoing spinal cord stimulation (SCS) therapy and understanding related complications and effects.

**Recent Findings:**

Litigation for SCS/DRG related complications can be very costly, with the mean average settlement in cases relating to SCS being $303,173 dollars in the USA. According to the US Anaesthesia Closed Claims Project database, 10,546 claims related to pain medicine were made between 1990 and 2013; 43 of which were associated with SCS complications. This article will further explore consenting and complications within the context of SCS.

**Summary:**

Consenting for SCS is a crucial and very important step which potentially reflect on patients’ expectations and outcomes.

## Introduction

Spinal cord stimulation (SCS) is a treatment, commonly used for management of several refractory chronic pain conditions, in particular; failed back surgery syndrome (now persistent spinal pain syndrome (PSPS type-2) [1, 2], complex regional pain syndrome (CRPS) [3], refractory angina (RA) [4, 5], chronic pains of trunk and limbs and critical limb ischemia [5, 6]. 

Other indications described for SCS include HIV and diabetic neuropathy [7], post-herpetic neuralgia, pain caused by central nervous demyelination such as multiple sclerosis etc [8]. 

Both NICE and the FDA have approved the use of SCS primarily for pain of neuropathic origin due to its reported effectiveness in a proportion of patients carefully selected using a multidisciplinary approach. However, the published literature is inconsistent, and the response rate is highly variable. Similarly, Dorsal root stimulation (DRG), another modality of neuromodulation has demonstrated a significant and clinically relevant effect on neuropathic painful conditions related to the trunk and limbs as well in cases of CRPS [9–11]. One trial has shown its superiority over SCS in the treatment of CRPS [12]. However, use of DRG is not without complications. Although some studies report fewer device related complications [13, 14], one RCT has reported higher complication rates compared to SCS [12]. 

Published evidence in a recent multi-centre setting demonstrates up to 81.4% responder rate when SCS was used to treat chronic lower back pain [15]. In a separate literature review carried out by Cameron et al. (2004), varied treatment success was attributed to phenotypic variability of painful conditions. Successful treatment rates included a 57% response rate in spinal cord injury, 62% in PSPS type-2 and phantom limb pain, 67% in peripheral neuropathy, 77% in ischaemic limb pain, 82% in post-herpetic neuralgia and 83% in CRPS I/II [16]. Similarly, a varied but consistent efficacy (42–82% pain relief) has been demonstrated for the DRG treatment across long term follow up [13, 14, 17]. 

Litigation for SCS/DRG related complications can be very costly, with the mean average settlement in cases relating to SCS being $303,173 dollars in the USA – almost double the average settlement for other claims associated with pain medicine [18]. According to the US Anaesthesia Closed Claims Project database, 10,546 claims related to pain medicine were made between 1990 and 2013; 43 of which were associated with SCS complications. During this period the use of implanted devices for pain management increased; alongside the proportion of pain medicine claims attributed to implantable devices, which increased from 3–16% [19]. As the use of SCS for chronic pain management increases, so does the importance of fully disclosing potential complications to patients; both from a clinical perspective and a medicolegal perspective. This article will further explore consenting and complications within the context of SCS.

## Shared Decision Making and Consent

The principle of shared decision making and consent to treatment is an important part of medical ethics, and fundamental to good medical practice. Patient consent is at the heart of good medical practice and patients have both a right to be involved in decisions regarding their treatment care and to be fully informed.

In order to facilitate informed decision making, patient concerns and expectations must be acknowledged, and they must be provided with clear and accurate information with adequate time for understanding and assimilation of said information. Clear exchange of information and communication between patients and doctors is important; as it helps manage expectations [20]. 

Before providing treatment or care for a patient, doctors must be satisfied that the patient has the capacity to consent. This entails being able to understand and retain information regarding treatment, using the information provided to settle on a decision and then finally communicating their decision. In all cases i.e. not just those involving issues of capacity; patients should be provided with ample time to ensure they understand the procedure and the risks involved, in order for them to be able to provide informed consent. If significant time has passed since the initial consent was given, it is the duty of the doctor to respond to any new or repeated concerns or questions of the patient before proceeding with the treatment [20]. 

Prior to treatment, doctors have a duty to make patients aware of any material risks involved in any recommended treatment, and of any reasonable alternative or variant treatments. A risk is material if a reasonable person in the patient’s position would be likely to attach significance to the risk. Therefore, doctors have a duty to inform patients of all potential significant adverse events that may occur as a result of said treatment; serious adverse events that have a low but well-established risk are likely to be regarded as significant by a patient. Awareness of these risks can have a large impact on a patient’s treatment preference, as they may choose not to undergo such treatment once they are made aware of such risks or choose to undergo a less risky and less efficacious treatment [20]. 

## Clinical Negligence, SCS/DRG and the Consenting Process

Clinical negligence can be defined as “an act of omission or commission in planning or execution that contributes or could contribute to an unintended result” [21]. 

This could involve giving substandard treatment or misdiagnosing a patient, causing injury to a patient or making an existing condition worse, giving incorrect or inappropriate treatment or making mistakes during surgery.

In the case of SCS/DRG this can manifest in a number of ways, for example by inappropriately offering SCS/DRG to patients for their presenting indications, by failing to obtain informed consent from patients by omitting reference to certain possible complications or by not explaining the chances of success and/or medical errors appropriately during device implantation management [18]. 

These instances of negligence can result in litigation by patients against the medical professionals involved in their SCS/DRG treatment. These claims often come with great financial and emotional cost for the doctor, but they also bear the non-monetary cost of patients losing confidence in the physicians responsible for their treatment.

## Screening and Complications Associated with SCS and DRG

SCS is not without risk of complications however there are many precautions that can be taken to reduce this risk, one of the most important being careful pre-implantation screening of the patients, as this often determines the success rate of the SCS/DRG therapy (Table 1).

**Table 1:** Patient screening criteria for SCS implantation outlined by the NACC (Neuromodulation Appropriateness Consensus Committee) [22]

Screening Criteria
• Patients must have capacity to give informed consent for the procedure
• Patients must agree to:
• Adhere to institutional protocol
• Attend post-implantation follow-up appointments
• Patients should have a well-defined, non-cancer, physiological source of pain:
• If the pain is related to surgical morbidity from tumour resection or cancer treatment and the patient is in stable condition, SCS may be considered
• The patient has undergone a 3–6-month trial of conservative medical management which has failed, additionally:
o The patient must not have undergone re-operation for back surgery
o The patient must be neurologically stable
o The patient must not be using long-acting opioid maintenance therapy
• The patient must possess sufficient cognitive ability to operate SCS/DRG equipment
• SCS/DRG should be avoided if remedial surgical procedures are not feasible, advisable or the preferred management for the patient
• The patient should undergo a well performed psychological screening in order to determine whether there are any major untreated or unstable psychiatric disorders; patients who are found to have significant somatisation complaints should be excluded.
• Expectations of the therapy should be set and discussed with the patient.
• The use of inappropriate drugs should be stopped prior to device implantation
• The patient must have a life expectancy greater than 12 months
• A preoperative MRI or CT myelogram of the spine should be carried out (< 12 months prior to the procedure) to rule out pathology that might confound diagnosis and/or compromise outcomes of SCS/DRG.
• The patient must not have any unresolved issues of secondary gain or litigation that could be central to the propagation of the pain complaint.

NICE (The National Institute of Health and Care Excellence) in UK has also published guidelines regarding the use of SCS. These guidelines recommend SCS as a treatment option for adults who have been suffering from chronic neuropathic pain for at least 6 months despite appropriate conventional medical management [23]. Examples of neuropathic pain include failed back surgery syndrome (PSPS type-2) [1, 2] and complex regional pain syndrome (CRPS) [3]. 

NICE does not recommend SCS as a routine treatment option for adults with chronic pain of ischaemic origin, except in the context of research as part of a clinical trial [23]. Examples of such conditions include refractory angina (RA) [4], chronic pains of trunk and limbs and critical limb ischemia [5, 6]. The aim of this research should be to generate robust evidence about the benefits of SCS in comparison to the standard care being offered for these conditions. However, if a patient is currently using SCS to treat chronic pain of ischaemic origin, they should have the option to continue treatment until they and their clinicians consider it appropriate to stop [23]. 

Furthermore, SCS/DRG should only be offered after an assessment by a multidisciplinary team (MDT) experienced in the assessment and management of chronic pain in patients who have been treated with SCS devices. A trial of SCS should be carried out as a part of this assessment; with the continuation of the treatment being dependent on its success. It is also important that this MDT contains individuals with experience in the provision of ongoing monitoring and support for patients receiving SCS treatment. When assessing the severity of pain and the trial of stimulation, the MDT should be aware of the need to ensure equality of access to SCS treatment. Tests to assess a patient’s pain and response to spinal cord stimulation should take into account any disabilities (e.g. physical or sensory disabilities), or linguistic or other communication difficulties, and should be adapted accordingly [23]. 

Following a recent multicentre RCT by Eldabe et al. (2020), a move towards direct to implant is prevailing based on nil significant advantage of trial versus direct to implant. Moreover, the study demonstrated cost-effectiveness and lower infection rate with single stage direct to implant procedures [24].

Spinal cord stimulation has been shown to be safe and reversible while also being effective in providing pain relief and improving functioning and quality of life in patients [25]. As is the case with many surgical interventions, spinal cord stimulation is associated with certain risks and complications. Approximately 30–40% of spinal cord stimulation patients may experience a range of complications that may necessitate some sort of intervention. The majority of these complications are minor and therefore reversible via minor interventions , meaning that they don’t usually impact patient morbidity or mortality in a significant way [22].

Complications of SCS/DRG can be grouped into hardware-related, biological and miscellaneous (Table 2). The most common hardware-related complications are lead related complications such as lead migration and fracture; these are correctable by lead replacement or repositioning. Superficial infection and pain over the implant side are the most common biological complications. Hardware related complications are more common than biological complications [22]. However, the technologies for SCS leads, IPGs, and programmers are rapidly evolving, resulting in fewer device-related events [26].

**Table 2:** A summary of complications associated with SCS and their rates of occurrence adapted from complications of spinal cord stimulation and peripheral nerve stimulation techniques: a review of the literature, Eldabe et al. [27]

Type of Complication	Complication	Rate of Occurrence (%)
Hardware/technology	Lead Migration	2.1 – 27% [28, 29]
	Lead Breakage	0 – 10.2% 33 [30, 31]
	Hardware Malfunction	2.9% [16]
	Battery/IPG Failure	1.6% [16]
	Loose Connection	0.4% [26]
Biological	Infection	2.5 – 10% [31, 32]
	CSF Leak	0–0.3% [27, 33]
	Epidural haematoma	0.3% [33]
	IPG Seroma	2.5% [22]
	Spinal Cord Injury	0.03–2.35% [32]
Other	Undesirable Stimulation	2.4% [16]
	Pain over implant	0.9 – 12% [16, 28]
	Skin erosion	0.2% [16]
	Allergic reaction	0.1% [16]
	Other	1.4% [16]

Historically, DRG has been associated with more common device related complications (lead fracture and displacement). Nevertheless, a recent safety analysis report published by Tim Deer et al. (2019) concluded DRG therapy event rates are similar to previously reported adverse event and complaint rates in the literature. Similarly, safety event rates were lower or similar to previously reported rates for SCS [34]. 

### Lead Migration

Lead migration remains the most prevalent hardware malfunction in SCS as well as DRG and tends to present as a change in the stimulation pattern for a patient. The angle of lead entry, battery placement as well as suturing techniques can all impact lead migration. Mechanical locking anchors have been shown to secure the leads with a higher tensile strength, adhesives have also been reported to improve anchor clinical performance, both of which decrease the likelihood of lead migration. Strain-relief loops near the anchor and IPG, and placement of the tip of the anchor into the supraspinous ligament can all contribute towards reducing lead migration. Ensuring the IPG is placed near the permanent leads can also reduce lead migration. A post-operative precaution that can be used to reduce lead migration is the limitation of a patient’s movements (especially those of twisting, bending etc.) during the post-operative period. This helps to scar the lead in the epidural space and decrease the amount it can migrate.

Lead migration can usually be visualised radiographically and should be compared to radiographs taken at the time of implantation. In order to remedy the migration, leads may have to be replaced or a paddle lead may have to be considered in certain cases, as they are less prone to movement than cylindrical percutaneous leads [22]. 

### Lead Disconnection/Fractures

Lead disconnection/fractures also present as a change in the stimulation pattern for the patient. The likelihood of lead fractures can be decreased by using stress loops and appropriate anchoring techniques, minimising distance between the IPG and permanent leads and also avoiding mobile structures such as joints. Macrofractures and lead disconnection from the IPG tend to be visible on x-ray whereas microfractures of a lead tend not to be visualised on x-rays. If there is any suspicion of micro- or macrofractures of the leads, impedance testing should be conducted in order to confirm it [22]. 

Should patients experience a change in stimulation pattern as mentioned above, re-programming should be attempted. If this fails then x-rays should be taken and compared to those done at time of implantation. There is a chance that these changes may be related to lead migration/fracture or potentially due to pre-existing or new neurological abnormalities in the patient. The solutions to changes in stimulation pattern are replacement or removal of the device and lead revision [22]. 

### IPG Failure

Batteries in IPGs can end up failing to hold charge or work properly. It is crucial that manufacturer instructions are followed carefully and that device connections are tested during implantation, as both can reduce the chances of battery/IPG failure. In order to rule out battery failure, the patient should be questioned about direct trauma to the battery site and x-rays should be carried out so that they can be compared to those taken at the time of implantation. In most cases battery failure will require replacement of the device, but prior to replacement it should be double checked with the manufacturer that this is not a known issue with the device in question [22]. 

### Infection

In cases of infection, patients may complain of any combination of nausea, vomiting, fevers, chills and malaise. Physical examination may reveal redness, warmth, swelling, pain and purulent drainage from the IPG site or any other incisions made during implantation. In cases of advanced infection, patients may also exhibit neurological changes.

Factors that increase risk of infection include immunosuppressive medications and conditions (e.g. corticosteroids, HIV, diabetes), prolonged hospital stay, undergoing multiple surgeries, perioperative transfusions, inappropriate or inadequate antibiotic therapy, poor ventilation of the operative suite and improper skin preparation at the surgical site. Certain comorbidities (e.g. diabetes, rheumatoid arthritis, malnutrition, obesity) and lifestyle choices (smoking tobacco, drinking alcohol) increase the risk of infection too [22]. Prophylactic antibiotics should be administered intravenously 30–60 min prior to surgery, with the exception of vancomycin which should be given within 120 min of surgery. The antibiotic dose should be calculated according to the patient’s body weight for maximum efficacy. The NACC recommends that antibiotics be discontinued within 24 h of surgery, however post-operative antibiotics may be considered in high-risk patients [35]. 

In addition to these other precautions used in high infection risk procedures should be utilised, such as limiting traffic through the operation theatre, irrigating wounds well and thorough scrubbing of the area.

If infection is suspected then FBC, ESR, CRP, wound cultures and blood cultures should be carried out. If the results point to a diagnosis of infection, then management usually involves opening of the device pocket and removal of the IPG. The IPG as well as the tissue and fluid cultures should be sent off to microbiology and pathology to identify the responsible pathogen and inform antibiotic choice [22]. 

### Post-Dural Puncture Headache (PDPH)

When performing epidural needle placement for lead positioning, an accidental dural puncture is a possibility. This can result in a patient experiencing a post-dural puncture headache (PDPH) as well as CSF leakage into the wound created by the needle. Patients who develop PDPH complain of positional headaches, diplopia, tinnitus, neck pain, photophobia. Most PDPH symptoms resolve within a week, some patients may require further intervention to get rid of them. Developing PDPH can complicate SCS trials for stimulation efficacy as the PDPH pain may become the focus of their pain rather than the indication for the SCS. Risk factors for dural puncture include uncooperative patients, calcification of the ligamentum flavum and previous surgery or spinal stenosis at the site of needle entry into the epidural space. If a patient experiences a dural puncture during their implantation, a range of opinions on management exist, where some surgeons may choose to abandon the procedure intraoperatively whilst others may continue at the same level or adjust the level of insertion. Conservative management such as supine positioning, caffeine and IV fluid administration may be used post-operatively in cases of dural puncture. Autologous epidural blood patches have also been used prophylactically and therapeutically in cases of dural puncture. However there is controversy surrounding their use as they may become a focus of infection later on [22]. 

### Epidural Haematomas

Epidural haematomas are a rare complication that may develop as a result of SCS trials or implantation. They tend to present with new neurological changes such as a paraesthesia not due to the SCS device; other possible symptoms include paralysis, weakness or bowel and bladder dysfunction. Other symptoms include radicular or back pain developing in a previously unaffected area or worsening of pain that is already present. The only risk factors that increase the chance of epidural haematoma are use of aspirin or other anticoagulant medication. It is important to follow the NACC recommendations on stopping these medications prior to implantation [36]. If epidural haematoma is suspected, an emergency CT scan with contrast should be carried out. If the findings correlate with that of an epidural haematoma, surgical evacuation of the haematoma should be carried out within 8 h of the initial neurological deficit. Prompt evacuation of the haematoma is crucial to reduce the risk of any neurological complications being permanent [22]. 

### IPG Seromas

IPG seromas present as redness, swelling and/or pain at the IPG site. Risk of seroma formation can be reduced by careful haemostasis and limiting the amount of blunt dissection and cautery done during implantation. Creating a smaller IPG pocket and using layered closure to limit dead space can also help to reduce seroma formation risk. Using an abdominal binder pre-emptively for approximately 2 months post-implantation is a technique that has been used sometimes to mitigate seroma risk. If an IPG seroma is suspected a CT with contrast should be carried out and the same blood tests mentioned in the previous paragraph should be conducted in order to rule out infection. Should a seroma form, it should be treated using an abdominal binder and drainage in a sterile environment, with wound cultures being sent off for analysis if infection is suspected [22]. 

### Contact Dermatitis, Allergic Reactions

Contact dermatitis, allergic reactions and foreign body reactions to SCS implants are known to happen in some instances. These may present as itching, malaise and/or pain around the implant site. If allergic reaction is suspected, blood tests should be carried out to rule out infection and skin patch testing can be carried out using a kit provided by the manufacture of the implant. It is important to bear in mind that the allergic reaction may in fact be due to other materials present during surgery, e.g. scrub solutions, sutures, medications, IPG, silicone, latex etc. If there is high pre-operative suspicion of allergic reaction to an implant, skin patch testing should be carried out prior to implantation. Implant removal may have to be considered in some cases, depending on the severity and cause of the allergic reaction [22]. 

### Pain Related to the Device Site

Patients may experience pain related to the site of device components, e.g. pain around the IPG site (pocket pain), lead anchor sites or lead extension junctions [22]. Incisional pain and scar tissue formation are believed to be contributing factors to pain over the IPG site. Pre-operative effectiveness of the endogenous analgesia system in a pain-free state and the intensity of acute post-operative pain are important predictors as to whether a patient will suffer from IPG pocket pain. Other factors that may affect the risk of developing pocket pain include pre-operative pain in the surrounding area, psychosocial vulnerability, younger age and female gender. Once low-grade infection and lead migration are ruled out as causes of pain, pocket pain is treated with conservative measures such as physiotherapy and medication for neuropathic pain. Should these conservative measures fail, the next solution is relocation of the IPG to a new site. Permanent removal of an IPG solely due to pocket pain is very rare and should be balanced against the gain from the therapy [37]. 

### IPG Flipping

IPG flipping occurs due to a large IPG pocket or as a result of Twiddler’s syndrome which involves patients manipulating the IPG within the pocket – it has an incidence of 0.54% [38–40]. This can result in twisting of the leads, breakage and inadequate IPG charging amongst other issues. The likelihood of IPG flipping can be decreased by ensuring the IPG pocket is of an optimal size and also by anchoring the IPG above the iliac crest subcutaneously in the lumbar region [39, 41]. 

### Skin Erosion

Skin erosion is a rare complication of SCS that tends to occur due to superficial placement of leads. IPG battery erosion or dehiscence can be avoided by placing the IPG away from bony sites and sites with high mobility. If a deep infection occurs to an SCS implant it should be removed regardless of whether the infection is systemic or not. After removal, MRI and wound cultures should be done to determine the presence of an epidural abscess. If re-implantation is going to be attempted precautions should be taken to avoid the same outcome and wound dehiscence [22]. 

### Nerve Root and Spinal Cord Injury

Some of the rare but serious complications of SCS implantation are nerve root and spinal cord injury. Spinal cord or nerve root trauma can be caused by a puncture from the introducer needle or it can be caused by the SCS implant leads. The symptoms of this can vary from no obvious injury, to sensorimotor deficits, to paralysis to neuropathic pain. In order to avoid this complication it is important that the surgeon implanting the device evaluates pre-operative imaging to determine important points of anatomy such as the location of the conus medullaris and any anatomical abnormalities that may increase the risk of trauma to spinal cord/nerve roots, e.g. spinal canal stenosis, spinal deformities and previous surgery at the site of needle placement [32]. 

### Explantation

Loss of therapeutic efficacy has been reported as the most common cause of explant in multiple studies. Recent technological breakthroughs have led to the availability of various waveforms and stimulation algorithms. These waveforms and stimulation algorithms provide promising outcomes by allowing the most suitable programs to be tailored to the needs of individual patients. However, these new technologies have their own limitations [38, 42], which must be weighed against the benefits they provide when consenting patients. Reliance on traditional paraesthesia based waveforms, unwanted stimulation and tolerance may have led to loss of efficacy in some cases. Literature suggests an explant rate ranging from 7.6–30% [43–45]. A recent survey from ASRA (American Society of Regional Anaesthetists) and SIS (Spine Intervention Society) members reports loss of efficacy as the primary reason for explantation of SCS devices (53.72%). Although loss of efficacy comprised a significant fraction of explants, the survey still contained significant reports of biological (12.2%) and device complications (20.7%) that necessitated removal [46]. 

## Summary and Conclusion

The paternalistic role of doctors deciding what treatment to offer a patient, which was unpinned by the decision in Bolam v Friern Hospital Management Committee [1957] 1 WLR 582 (where the standard of care required of a doctor was judged by the standard to be expected of a reasonable group of their peers) is no longer acceptable and does not hold value in modern day clinical practice [47]. 

The emphasis is now on a patient’s choice made after being informed of clinically appropriate options. The patient has the right to choose a therapeutic option and its possible impact on their health. The medical advice about treatment options must consider medical factors whilst taking into account the patient’s value judgements. The implications relate to the clinicians necessitating to familiarise themselves with their patients sufficiently well to understand their views and values and thereby support them in the decision-making process.

The acceptable standard of patient / clinician interaction in the consenting process is now much higher. The assessment of that standard now will combine factors important to both the clinician and patient. Clinicians should ensure there is enough space and time allowed for jargon-free, meaningful, patient-centred dialogue. A short conversation, just prior to surgery, with an unfamiliar patient pulled from a group waiting list is unlikely to reach that standard.

Providing patients with evidence-based information and realistic expectations of SCS treatment outcomes would bolster patient confidence in the clinicians delivering SCS treatments; and allow them to be reassured that their decisions regarding treatment are based on a foundation of accurate and up-to-date information.

Although there are many clear and thorough guidelines describing the nuances of SCS device implantation and patient selection, no such guidelines exist on consenting patients for SCS, leaving physicians unsupported in this process and therefore at risk of litigation. This could relate to poor patient understanding of various risks and expectations that were set during their consenting for implantation. Moreover, in the absence of insufficient data on the efficacy of SCS in socioeconomically deprived populations, consenting of this subgroup could be challenging and would require a judicious approach in order to avoid precluding these groups when offering therapy [48]. This article emphasises the importance and need for such guidelines to be developed, in order to ensure that informed consent is gained satisfactorily and that patient expectations are managed appropriately, with the aim to ensure the highest standards of care and to maintain the integrity of both the care provider and the therapy being provided. A standardised consenting paradigm can help in developing confidence among clinicians and patients by minimising undue litigation risk significantly.

## Key References

26. Kumar K et al. (2007) Avoiding complications from spinal cord stimulation: Practical recommendations from an international panel of experts. Neuromodulation: Technology at the Neural Interface. 2007;10(1):24–33. 10.1111/j.1525-1403.2007.00084.x. *complementary article outining complications.

34. Deer T, et al. Safety analysis of dorsal root ganglion stimulation in the treatment of chronic pain. Neuromodulation: Technol Neural Interface. 2020;23(2):239–44. 10.1111/ner.12941. ** recent article discussing safety of dorsal root ganglion implants.

## Data Availability

No datasets were generated or analysed during the current study.
